# Linking diet and obesity among young people living in Sub-Saharan Africa: a systematic review

**DOI:** 10.3389/fnut.2026.1859486

**Published:** 2026-07-21

**Authors:** Chidiogo R. Ajuluchukwu, Favour C. Ogu, Kosisochukwu C. Igbokwe, Uju M. Onuorah, Beulah F. Ortutu, Gideon O. Iheme

**Affiliations:** 1 Department of Human Nutrition and Dietetics, Michael Okpara University of Agriculture, Umudike, Nigeria; 2 Department of Human Nutrition and Dietetics, University of Ibadan, Ibadan, Nigeria; 3 Department of Nutrition and Dietetics, University of Nigeria, Nsukka, Nigeria; 4 Department of Nutrition, Texas A & M University, College Station, TX, United States; 5 Department of Food Studies, Nutrition and Dietetics, Uppsala University, Uppsala, Sweden

**Keywords:** adolescents, children, diet-related practices, overweight and obesity, Sub-Saharan Africa

## Abstract

**Background/objectives:**

Obesity has been increasing even among young people in developing countries, with unhealthy diets widely recognized as a major contributor to the rising risk of obesity and other non-communicable diseases (NCDs). This review aimed to assess the relationship between diet-related practices and obesity amongst young people in Sub-Saharan Africa.

**Methods:**

A review protocol was developed *a priori* and registered with PROSPERO (CRD42023451525). This study followed PRISMA guidelines to search and select studies based on pre-defined eligibility criteria from African Journals Online, PubMed, and Google Scholar databases. Out of 2,809 screened citations, data were extracted from 19 eligible peer-reviewed studies published in English between 2000 and 2024. These studies focused on children and adolescents aged 3–19 years and examined the association between dietary habits or practices and elevated anthropometric status.

**Results:**

Results showed that the selected studies were cross-sectional in design, typically conducted among fewer than 500 children from Nigeria (31.6%), Ghana (15.8%), and South Africa (15.8%). Food frequency questionnaires, 24-h recall instruments, the WHO growth reference, and Waist-Hip assessment dominated dietary pattern/intake and anthropometric methods. Negative dietary patterns/habits characterized by meal skipping and irregular meal patterns, discretionary food and beverage consumption, less-varied diets, and unhealthy urban/school food environments were significant (*p* < 0.05) predictors of obesity.

**Conclusion:**

Dietary intake was significantly associated with Obesity. Promoting healthy diet-related practices is needed to improve children and adolescents’ nutrition and health outcomes.

**Systematic review registration:**

https://www.crd.york.ac.uk/PROSPERO/view/CRD42023451525.

## Introduction

Obesity is a global public health problem (1). One out of every five children and adolescents is either overweight or obese (1). Although the prevalence of obesity is highest in developed countries, where about a third of the children are overweight/obese (2), evidence has shown that the African continent is home to a quarter of all overweight children in the world (3). Moreover, the burden of overweight and obesity has risen dramatically over the last four decades, as developed and developing countries recorded an increase from 16.2–16.9% and 8.1–8.4% to 22.6–23.8 and 12.9–13.4% between 1980 and 2013, respectively (4).

The predictive linkages between childhood, adolescent, and adult obesity have been established in the literature (5, 6). Obese children and adolescents are five times more likely to be obese in adulthood than their non-obese counterparts (6). Obesity in early life years has been implicated in the emergence of non-communicable diseases and mortality in adulthood (7). Overweight and obesity are the fifth major cause of global death, accounting for over 2.8 million deaths every year (8).

The evolving African food environment (9–11), decrease in global out-of-school rates albeit stagnant figures reported in Sub-Saharan Africa and higher number of awake hours spent in school than home (12, 13), busy work and study schedules of parents and children, respectively, as well as other external influences like peer pressure and the media (14, 15), are likely to affect the dietary habits and overall lifestyle of children and adolescents which have been touted as the main modifiable risk factors of Obesity. Several pooled evidence reviews have examined dietary habits or food consumption in children/adolescents and their associated obesity risks. However, some of these studies were not recent (16, 17), covered only Asia (16), and middle east regions (18), exempted specific African focused databases (16, 17, 19), or focused on obesity emergence later in life (17), In order to stimulate policy and programmatic action to improve dietary patterns and their impact on nutrition status in low-income settings, this study was designed to pool evidence on the prevailing dietary-related practices and weight status of children and adolescents in Sub-Saharan Africa and their interconnections.

## Methods

A review protocol was developed *a priori* and registered with PROSPERO (CRD42023451525) to enhance methodological transparency. This systematic review was reported in accordance with the PRISMA statement (20). The completed PRISMA 2020 checklist, including the location of each reporting item within the manuscript, is provided in Supplementary file 2.

### Search methods for the identification of studies

A systematic literature search was conducted between November 2024 and April 2025 in PubMed, African Journals Online (AJOL), and Google Scholar databases to identify peer-reviewed original articles published between 2000 and 2024. The search strategy was developed in PubMed using a combination of Medical Subject Headings (MeSH) and free-text terms related to dietary habits, dietary patterns, nutrient intake, obesity, overweight, weight gain, and children and adolescents in Sub-Saharan Africa, combined using Boolean operators (“AND” and “OR”). This strategy was then adapted to the indexing systems and search functionalities of AJOL and Google Scholar. The complete PubMed search strategy is provided in the Supplementary file 1 (Table 1).

**Table 1:** Inclusion and exclusion criteria.

S/No	Inclusion criteria	Exclusion criteria
1.	Studies involving children and adolescent aged 3–9 years	Studies involving adult (>19), elderly or infant populations
2.	Studies conducted in countries within Sub-Saharan Africa country	Studies conducted outside Sub-Saharan Africa
3.	Peer-review original research employing observational study designs (cross-sectional, cohort or case–control)	Review articles, editorials, commentaries, conference abstracts, case reports, letters, dissertations, and other grey literature
4.	Studies published in English between 2000 and 2024	Studies not published in English or outside the specified publication period
5.	Studies examining dietary intake, dietary patterns, eating habits, meal skipping, breakfast consumption, fast-food consumption, nutrient intake, or related dietary exposures	Studies that did not assess diet-related or nutrition-related exposures
6.	Studies reporting overweight and obesity through elevated BMI-for-age, waist circumference, waist-to-hip ratio (WHR), waist-to-height ratio (WHtR), body fat measures outcomes	Studies that did not report overweight and obesity-related outcomes

### Study selection and inclusion criteria

Two reviewers independently screened all titles and abstracts of the peer-reviewed publications to ensure adherence to the defined eligibility criteria. Full-text versions of potentially eligible articles were subsequently obtained and independently reviewed for inclusion. Additionally, the reference lists of selected articles were checked to identify relevant studies not captured during the electronic database search. Any discrepancies or disagreements regarding the inclusion of studies were resolved through discussion with the entire team. The inclusion criteria were formulated according to the PEOS (Population, Exposure, Outcome, Study Design) framework.

Population: Young people (children and adolescents) in Sub-Saharan Africa aged 3 to 19 years were included.
Exposure: The exposure of interest included dietary patterns, dietary intake, meal frequency, meal skipping, discretionary food consumption, dietary diversity, alcohol consumption and breakfast consumption.
Outcome: Primary outcomes included overweight, obesity, BMI-for-age, waist-to-hip ratio (WHR), waist-to-height ratio (WHtR), adiposity, and anthropometric indicators defined according to WHO. Obesity and weight gain were evaluated using all forms of anthropometric measurements.
Study design: Observational studies (cross-sectional, cohort, and case–control) published in peer-reviewed journals between 2000 and 2024 were eligible for inclusion if they examined the association between dietary habits, dietary patterns, or nutrient intake and weight gain or obesity.

### Data extraction

Data were extracted manually from the included studies using standardized data extraction tables developed by the review team. Information extracted included study characteristics (e.g., author, year of publication, country, study design, sample size, and participant characteristics), dietary exposures, obesity-related outcomes, and their associations as well as other assessment methods, and key findings relevant to the review objectives. Two reviewers independently extracted and cross-checked the data to ensure accuracy and consistency. Any discrepancies were resolved through discussion and consensus among the review team. No automation tools were used during the data extraction process.

### Risk-of-bias assessment

Two reviewers independently assessed the risk of bias, and any discrepancies resolved by the team. The selected studies were rated using a modified version of the Joanna Briggs Institute (JBI) critical appraisal tool for analytical cross-sectional studies (21).

The original checklist was adapted to better align with the objectives and characteristics of the included studies. Specifically, items focused on identifying and addressing confounding factors were removed, owing to limited casual inference nature of the selected studies. Consequently, the modified checklist comprised six appraisal criteria.

The six criteria assessed were: (1) criteria for inclusion in the sample clearly defined, (2) study subjects and settings description, (3) exposure measured in a valid and reliable way, (4) objective, standard criteria used for measurement of condition, (5) outcomes measured in a valid and reliable way and (6) appropriate statistical analysis, using the ‘yes,’ ‘no,’ ‘unclear,’ and ‘NA’ (non-applicable) option. Studies with insufficient information to judge a criterion were classified as Unclear. All discrepancies were resolved by the authors.

### Sub-group analysis

Data from this study were primarily grouped to provide information on key diet-related practices that shape high anthropometric outcomes. These were subdivided into meal skipping, discretionary food and beverage consumption, and school food environment. Other factors affecting dietary patterns/habits, or nutritional status, were also evaluated. The summary statistics for the relevant participant and study characteristics were computed and presented, and a *p* < 0.05 was considered significant for establishing the association between diet and obesity in selected studies. All analysis were done using IBM SPSS Version 27.

## Results

**Figure 1:**
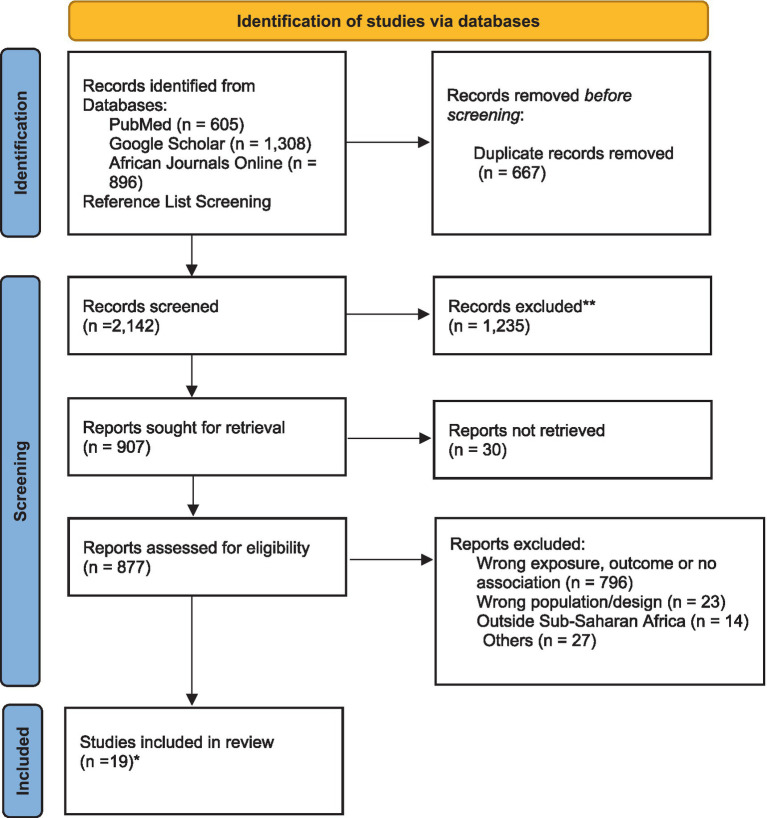
PRISMA flow chart of article selection. * Two included studies were identified through manual reference-list screening. As the number of records retrieved through this process was not recorded, they are reported only at the final inclusion stage of the PRISMA flow diagram.

A total of 2,809 records were identified through searches of the three databases, covering publications from 2000 to 2024. After the removing duplicates, 2,142 records remained for screening. Title and abstract screening were conducted independently according to the predefined eligibility criteria, resulting in the exclusion of 1,235 records. The remaining 907 articles were sought for full-text review; however, 30 articles could not be retrieved because the full texts were unavailable or inaccessible. Consequently, 877 full-text articles were assessed for eligibility. Following full-text screening, 860 articles were excluded, primarily because they reported ineligible outcomes, did not assess relevant dietary exposures, or did not examine the association between dietary factors and obesity-related outcomes. Seventeen (17) studies met the eligibility criteria during the database search. In addition, manual screening of the reference lists of relevant articles identified two further eligible studies. Ultimately, 19 peer-reviewed studies met the inclusion criteria and were included in the review (Figure 1).

### Description of included studies

A total of 19 studies were included; a good number (31.6%) of the studies were conducted in Nigeria, less than a quarter (21.1%) were conducted in Ethiopia, while a few (15.8 and 15.8%) were conducted in Ghana and South Africa, respectively, with the majority (94.7%) having a cross-sectional study design. These studies were carried out from 2000 to 2024 across various African cities and countries. The included studies (89.5%) cover the period from 2014 to 2024. The majority (73.7%) of the studies were school-based, while a quarter (21.1%) were conducted in urban and rural areas in different cities in the country. The studies in this review collectively targeted 11,394 individuals. About one-fifth (21.1%) of the studies had a sample size exceeding 1,001, and 21.1% had a sample size of 601 to 700, respectively. Less than half (44.4%) of the studies used multistage sampling, while some (33.3%) used random sampling techniques.

Included studies employed a combination of validated standardized instruments and researcher-developed questionnaires, which were either validated or non-validated (36.8%). These included Food Frequency Questionnaires (21.1%), 24-h dietary recalls, FAO dietary diversity tools, and structured dietary behaviour questionnaires. In comparison, other studies used fewer dietary pattern/intake instruments (7.14%) to assess participants’ dietary patterns. About a quarter (26.3 and 21.1%) of the studies used the WHO reference standard/Anthroplus software, and the WHO reference standard, respectively, while a few (10.5 and 10.5%) studies used the WHO reference standard/WHR/WHtR and IOTF, respectively, to assess anthropometric measurements.

About a quarter (26.3 and 21.1%) of the studies used the Chi-square test and Fisher’s exact test, respectively, while over a tenth (15.8%) used binary and multiple logistic regression. Detailed characteristics of each study are presented in Table 2, and a brief descriptive summary is provided in Table 3.

**Table 2:** Characteristics of Included Studies.

Authors	Country/location	Study population	Sample size	Study design	Sampling technique	Dietary pattern/intake instruments	Anthropometric indices	Other variables
Kirsten et al. (22).	South Africa- Stellenbosch area/Western Cape province	School-aged children (6–13 years)	638	Cross sectional, comparison study	Randomly sampling	Questionnaire – Eating behaviour Food frequency questionnaire– food consumption	Measurements – Weight and Height Indicator – International obesity task force	Socio-demographic and family characteristics Lifestyle
Teshomeet et al. (23)	Southern Ethiopia – Hawassa	Adolescents (10–19 years)	599	Cross sectional study	Stratified sampling	Food frequency questionnaire– frequency of food consumption	Measurements – weight, heights and skinfold thickness Indicator – BMI for age (WHO growth reference 2007) WHO Anthro-Plus	Demographic information Socio-economic Physical activity
Gebremichaelet al. (24)	Ethiopia– Addis Ababa	School-aged children and adolescents (10–18 years)	463	Cross sectional study	Multi-stage sampling	Questionnaire– eating habit	Measurements – Weight, Heights Indicator – BMI for age (CDC growth charts)	Socio-demographic Physical activity
Lateef et al. (25)	Nigeria-Kwara state	School-aged children and adolescents (5–19 years)	515	Cross sectional	Multistage random sampling	24 h recall and Food frequency questionnaire (FFQ) – breakfast and food consumption	Measurements – Weight and Height Indicator – BMI for age (WHO growth reference 2007) Anthroplus software	Demographics characteristics
Otuneye et al. (26)	Nigeria-Abuja municipal	Adolescents (10–19 years)	1,550	Cross sectional study	Multistage stratified sampling	Self-designed questionnaire – dietary habits and knowledge of nutrition	Measurements – height, weight BMI Indicator – BMI for age (WHO growth chart 2007)	Socioeconomic status General physical examination
Sedibe et al. (27)	South African – Agincourt (rural) and Soweto (urban)	Early adolescents and mid-adolescents (13 and 15 years)	3,490	From Health and Demographic Surveillance System (AHDSS) and Birth to Twenty (Bt20)	–	Questionnaire – Dietary Habits and Eating Practices Assessment	Measurements – weight and height Indicator – BMI for age (WHO growth reference 2007) AnthroPlus 2007	–
Alangea et al. (28)	Southern Ghana-Ga-East Municipality	School-aged children and adolescents (9–15 years)	487	Cross sectional study	Random sampling	A 7-day food frequency questionnaire (FFQ) – food frequency and dietary patterns	Measurement – height and weight Indicator – BMI for age and sex (BMA) WHO AnthroPlus	Demographic characteristics
Tluway et al. (29)	Northern Tanzania/Babati District	School-aged children and adolescents (10–19 years)	619	Cross sectional survey	Multi-stage cluster sampling Randomly sampled Probability proportion-to-size	Food Frequency Questionnaire (FFQ) – dietary habits	Measurements – weight, height and BMI Indicator – International Obesity Task Force (IOTF)	Socio-demographic characteristics Physical activity Sedentary behaviour
Gyamfi et al. (30)	Ghana–Ashanti-Bekwai Municipality.	School-aged children and adolescents (5–17 years)	1,004	Cross-sectional study	Random sampling	A questionnaire – eating habits (snacking status)	Measurements – weight, height, waist circumference and hip circumference (WHR) Indicators – BMI-for-age (WHO growth reference 2007) Waist and hip ratio (WHR) Waist-to-height ratio (WHtR)	Demographic characteristics – the parents and caregivers Physical activity
Annan et al. (31)	Ghana-Kumasi	School-aged children (8–13 years)	438	Cross-sectional study	Random sampling	FAO’s nutritional practices questionnaire– breakfast consumption pattern	Measurements – Weight, Height, MUAC Indicators– BMI for age (WHO growth reference 2007) WHO Anthroplus software	Physical fitness
Adeomi et al., (32)	Nigeria- Lagos	Adolescents (10–19 years)	260	Cross sectional study	Two-stage sampling technique Purposive sampling Random sampling technique (balloting method)	Questionnaire–Frequency of food consumption Dietary diversity score–24-h dietary recall	Measurements – weight, heights, stunted, wasted, underweight Indicator – BMI-for-age (WHO growth reference 2007)	Socio-demographic characteristics Physical activity patterns
Debeila et al. (33)	South Africa – Fetakgomo Municipality/Limpopo Province	Adolescents (13–19 years)	370	Cross sectional study	Multistage sampling	A set of 10 true or false questions – nutrition knowledge and food frequency questionnaire– dietary practices	Measurement – weight, height, waist circumference, hip circumferences Indicator – International Obesity Task Force (IOTF) Waist-to-hip ratio (WHR) Waist-to-height ratio (WHtR)	Socio-demographic factors
Tunkara-Bah et al. (34)	Gambia–Banjul and Kanfing Municipal	In-school adolescents (13–19 years)	1,008	Cross sectional study	Multi-stage sampling	Questionnaire – dietary habits	Measurement– weight and height Indicator – (WHO growth reference 2007)	Demographic information
Adeomi et al. (35)	Southwestern Nigeria – Ile-Ife	In-school adolescents (10–19 years)	400	Cross sectional	Multi-stage sampling technique	Frequency questionnaire (FFQ) – dietary patterns	Measurements – weight and height Indicator – International Society for the Advancement of Kinanthropometry	Socio-demographic Physical activity Lifestyle
Mapfumo et al. (36)	Zimbabwe-Harare	Adolescents (14–16 years)	111	A cross-sectional study	Systematic random sampling	FAO adapted questionnaire–Nutrition knowledge, dietary pattern and dietary diversity (DD)	Measurements – heights, weight, waist circumference and hip circumference Indicator – BMI for age (WHO growth reference 2007) Waist to hip ratio (WHR) Waist-to-Height to hip ratio (WHtR)	Demographics data Physical activity Blood glucose Blood pressure measurements
Olatona et al. (37)	Southwestern Nigeria – Lagos State	Adolescents (10–19 years)	682	Cross sectional study	Multi-stage sampling method	Questionnaire – nutritional knowledge Food frequency questionnaire – dietary habits 24 h dietary recall – nutrient intake	Measurement – weight and heights Indicator – BMI for age (WHO standard)	Socio-demographic
Samuel et al. (38)	Nigeria – Owo LGA, Ondo State	School-aged children and adolescents (10–18 years)	1,000	Cross sectional study	Multistage sampling	Questionnaire – dietary pattern/alcohol consumption	Measurements – weight, height, skinfold thickness, Waist circumference and hip circumference Indicator – BMI for age [Center for Disease Control and Prevention (CDC) chart] Waist-to-hip ratio (WHR) Waist-to-ratio (WHtR)	Demographic information
Biadgilign et al. (39)	Ethiopia–Addis Ababa	School-aged children and adolescents (5–18 years)	632	Cross sectional study	Multi-stage sampling	24-h dietary recall – dietary intake Dietary diversity score (DDS)	Measurements – weight and height Indicator – BMI for age (WHO growth reference 2007) WHO Athroplus	Socioeconomic status/wealth index Socio-demographic
Elias et al. (40)	Ethiopia–Wolaita Sodo	Adolescents (10–19 years)	563	Cross sectional study	Random sampling	Questionnaire – dietary information Habits of fast-food consumption	Measurements – weight, heights, waist circumference and hip circumference Indicator – BMI for age (WHO growth reference 2007) WHO Anthro-plus Waist-to-hip ratio (WHR)	Socio-demographic physical activity Sedentary lifestyle characteristics Alcohol intake

**Table 3:** Descriptive summary of study characteristics.

Variables	Frequency (F)	Percentage (%)
Country/location		
Nigeria	6	31.6
Ghana	3	15.8
South Africa	3	15.8
Ethiopia	4	21.1
Tanzania	1	5.3
Zimbabwe	1	5.3
Gambia	1	5.3
Publications year		
2000–2013	2	10.5
2014–2024	17	89.5
Study setting		
School	14	73.7
Urban/rural area	4	21.1
Orphanage	1	5.3
Sample size		
101–200	1	5.3
201–300	1	5.3
301–400	2	10.5
401–500	3	15.8
501–600	3	15.8
601–700	4	21.1
901–1,000	1	5.3
1,001 and above	4	21.1
Study design		
Cross sectional study	18	94.7
Health and demographic surveillance system	1	5.3
Sampling technique		
Random sampling	6	33.3
Multistage sampling technique	8	44.4
Two stage sampling + probability proportional sampling	1	5.6
Random sampling + multistage sampling technique + probability proportion sampling	1	5.6
Stratified sampling	1	5.6
Random sampling + two stage sampling + purposive sampling	1	5.6
Dietary pattern/intake instruments		
Self designed questionnaire	7	36.8
FAO’s nutritional practice questionnaire	2	10.5
A set of true/false questions/FFQ	1	5.3
FFQ	4	21.1
Self designed/24 h/DDS	1	5.3
Self designed/FFQ/24 h	1	5.3
Self designed/FFQ	1	5.3
24 h/FFQ	1	5.3
24 h/DDS	1	5.3
Anthropometric indices		
WHO reference standard	4	21.1
WHO reference standard/WHR/WHtR	2	10.5
WHO reference standard/WHR/Anthrroplus software	1	5.3
WHO reference standard/Anthrroplus software	5	26.3
IOTF	2	10.5
IOTF/WHR/WHtR	1	5.3
CDC	1	5.3
CDC/WHR/WHtR	1	5.3
International Society for Advancement of Kinathropometry	1	5.3
WHO Anthroplus	1	5.3
Statistical test for association		
Chi-square test	5	26.3
Pearson’s correlation + ANOVA	1	5.3
T-2 independent sample *t*-test	1	5.3
Chi-square test and Fisher’s exact test	3	15.8
Binary logistic and multiple logistic analyses	4	21.1
Bivariate and multivariate linear regressions	1	5.3
Chi-square and Pearson’s correlation	1	5.3
Chi-square, bivariate analyses, multivariate logistic regression	2	10.5
Chi-square, Student’s *t*-test and binary logistic regression	1	5.3

### Quality assessment of included studies

**Figure 2:**
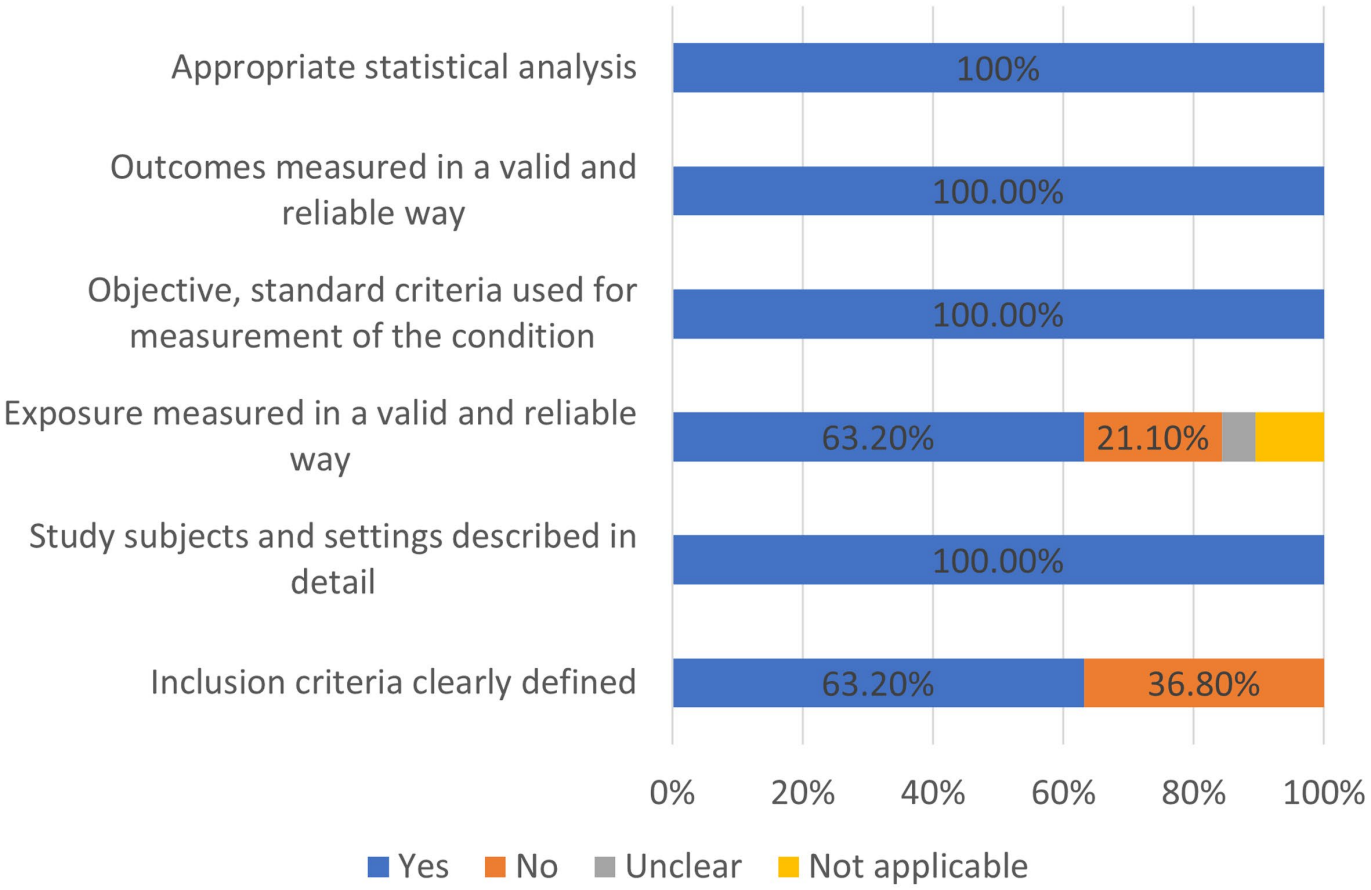
Quality assessment of included studies.

The quality assessment/appraisal using the JBI critical appraisal checklist for descriptive/case studies, as presented in Figure 2, showed that all included studies (100.0%) clearly described their study populations and settings, employed appropriate statistical analyses, and assessed obesity-related outcomes and conditions using objective, standardized, and valid measurement methods. However, inclusion criteria were not clearly defined in over one-third (36.8%) of the selected studies. Similarly, some studies fell short of using a reliable and valid approach to measuring diet-related exposure.

In general, these studies had good overall quality, with scores ranging from 4 to 6 out of a possible 6 criteria, suggesting a low risk of bias and good methodological quality relevant to this study. Details of the assessment of the individual components of the risk of bias can be found in Supplementary file 1 (Table 2).

### Dietary patterns and obesity

The studies (22–40), under review, have underscored that the consumption of an unhealthy diet is a significant factor responsible for the problem of overweight levels among children and adolescents (Table 4).

**Table 4:** Diet and obesity linkages.

Author	Diet variables	Overweight and obesity	Association
Kristen et al. (22)	Breastfeeding Majority (84%) of the children were breastfed	Body Mass Index (IASO/IOTF) Overweight – 9% Obesity – 4%	Chi-square No significant association was found between breastfeeding practices as a protective factor in the prevention of childhood overweight or obesity.
Teshome et al. (23)	Dietary pattern (breakfast consumption, fruits and vegetable, fast food, meat, and consumption) More than half (50.4%) of the respondents never skipped their breakfast. Minority of participants used vegetables (28.7%) and fruit (27.6%) only three to six times per week. Only few participants consumed meat (17.8%), and fast food (20.2%) either more than once per day or once per day.	Body Mass Index for age (WHO Anthro-plus) Overweight – 12.9% Obesity – 2.7%	Chi-square and multiple logistic regression No association regarding the effect of breakfast skipping and prevalence of overweight and obesity. Adolescent students who eat fruit twice per month or less are 4.67 times more likely to be overweight than adolescents who eat fruit more than two times per day (AOR = 4.67 [95%CI: 1.76–12.38]). The odds of being overweight were 91% lower in adolescents who eat meat twice per month or less compared with adolescents who eat meat once or more than once per day (AOR = 0.09 [95%CI: 0.03–0.25]). The odds of being overweight were 88% lower in adolescents who eat fast food twice per month or less in the previous month compared with adolescents who eat fast food more than once per month (AOR = 0.12 [95%CI: 0.04–0.34]).
Gebremichael et al. (24)	Breakfast consumption About (32%) of the respondents eat breakfast regularly Snack consumption Majority (70.2%) of the respondent ate snacks once a day Few ate snacks mainly twice or three times a day (14.5 and 1.9%).	Body Mass Index for age Overweight – 9.9% Obesity – 2.8%	Logistic regression Eating breakfast regularly was found to be protective for overweight. There is a significant association between irregular consumption of breakfast and overweight Eating snacks has a strong association with being overweight. The odds of becoming overweight from eating snacks 2–3 times per day is 10–26 times higher compare to eating 1 time a day
Lateef et al. (25)	Food consumption pattern Breakfast consumption Majority (77%) of the participants ate breakfast before going to school while 23% skipped breakfast daily	Body Mass Index for age (WHO Anthro-plus) Overweight – 4.7% Obesity – 0.2%	Pearson correlation Relationship between participants food consumption pattern and nutritional status was weak and of no significance (*r* = 0.012, *p* = 0.785) ANOVA There is a significant association between the categories of nutritional status and their food consumption pattern of participants
Otuneye et al. (26)	Meals intake (skipping meals) The skipping of main meals was prevalent by 9.5% Some of the respondent skipped breakfast (4.6%), lunch (3.8%) and supper (1.1%)	Body Mass Index for age Overweight – 12.6% obese – 2.8%	Chi-square No significant association between skipping meals and overweight and obesity
Sedibe et al. (27)	Breakfast consumption More than half of the two categories of respondents (early and mid-adolescent) in both cities consumed breakfast and snacks during the week (60%). A good number of both categories of respondents (urban and rural early-adolescents) consumed breakfast irregularly (25% vs. 8.87%).	Body Mass Index for age (WHO Anthro-plus) Overweight; 12.6–13.8% Obesity; 8.8–8.9%	Logistic regression Irregular frequency of breakfast consumption on weekdays were associated with increased risk of being overweight and obese among (early and mid-adolescents).
Alangae et al. (28)	Dietary patterns A good number of the respondents consumed energy dense food and starchy roots and vegetables (21.6 and 13.7%) while few consumed cereal-grain and poultry (10.4 and 7.4%) The frequently consumed energy dense foods were sugar sweetened beverages, candies, ice creams, fried foods, processed meats, spreads, toppings, and snacks like cakes, pies, doughnuts and other savory foods.	Body Mass Index For age (WHO Anthro-plus) Overweight/obese – 17.7%	Simple linear regression There was a significant association between consumption of energy dense diet and child overweight status [*F*(5, 479) = 6.868, *p* < 0.001]. Starchy root with vegetable DP was negatively associated with overweight/obese status [*F*(2, 482) = 7.369, *p* = 0.001]. Relationship between ‘starchy root staples and vegetables’ DP and overweight/obese status lost significance after controlling for other covariates Multiple logistic regression There was a positive association between energy dense pattern and child overweight (AOR = 1.34, 95%CI = 1.04–1.70). Childhood overweight was not significantly associated with any of the other three dietary patterns.
Tluway et al. (29)	Unhealthy diet Majority (86.2%) of the respondents consumed unhealthy diet	Body Mass Index for age Overweight/obese – 9.2%	Chi-square Unhealthy diet has been associated with diet higher prevalence of overweight or obesity (*χ*^2^ = 13.8, *p* = 0.017) Multiple regression Unhealthy diet increased the odds of being overweight or obesity by a factor of two (AOR = 2.2; 95% CI: 1.32, 3.63).
Gyamfi et al. (30)	Snack consumption timing (in-between meal); More than half of the two categories of respondents (elementary and high school participants) consumed snacks before breakfast (58.7% vs. 55.3%). A good number also ate snacks mainly between lunch and super intervals (27.5% vs. 20.4%).	Body Mass Index for age (WHO Anthro-plus) Overweight: 12.6–13.8% Obesity: 8.8–8.9% Waist to height ratio Obesity: 5–10%	Cross tabulation and chi-square: Overweight and obesity was statistically more prevalent amongst snack consumption (42.7 and 23.1%) before bedtime compared to other meal intervals (4.7–7.9%) Logistic regression The odds of becoming obese/underweight from snacking before bedtime was >10 times higher
Annan et al. (31)	Breakfast consumption Majority (78.9%) of the pupils consumed breakfast, that is, ate before coming to school	Body Mass Index for age (WHO Anthro-plus) Overweight – 9.4% Obesity – 1.1%	Chi-square No association between breakfast intake and BMI status (*χ*^2^ = 0.362, *p* = 0.865)
Adeomi et al. (32)	Dietary diversity (food frequency consumption) Majority of the respondents consumed fruits (97.7%), animal proteins (96.2%), carbohydrates (96.2%), plant proteins (95.4%), eggs (92.7%) and vegetables (80.0%), more than 3 times	Body Mass Index for age (WHO Anthro-plus) Overweight − 5.4% Obesity – 3.5%	Chi-square The relationship between nutritional status and dietary diversity was not statistically significant (*χ*^2^ = 0.609, df = 2 *p* = 738)
Debeila et al. (33)	Meal intake (breakfast) Majority of the respondents consumed breakfast (74%), lunch (66%) and supper (78%) daily Common breakfast foods include; bread, cereals and porridge	Body Mass Index (IOTF) Overweight – 26.0% Obesity – 9.0% Waist circumference Abdominal obesity – 9% Waist hip ratio Abdominal obesity – 25% Waist to height ratio Abdominal obesity – 21%	Multiple logistic regression Eating breakfast accounted for lower odds of being overweight/obese (AOR = 0.6, 95% CI: 0.34–0.97).
Tunkara-Bah et al. (34)	Dietary factors Sugar and sweetened drinks Majority of the students (72.3%) drank soft drinks in the preceding month of data collection while some (23.5%) drank soft drinks month before the data collection. Majority (90.2%) of the students drank a sugar added juice. Dairy foods and oils Most of the students (37.9%) eat bread spread with mayonnaise at least once a day. Almost half of the students (43.3%) said they did not know the different types of milk but (24.9%) used whole or full cream milk. A good number (40.1%) ate fried food daily. Many of the participants ate sweets such as candy two to four times a week (3.2%) and (25.8%) of the participants ate sweets more than four times per week. More than half of them (57.4%) ate biscuits or cakes as snacks	Body Mass Index for age (WHO Anthro-plus) Overweight – 7.7% Obesity – 0.7%	Chi-square The frequency of drinking sugar-added juices was significantly associated with overweight and obesity among the participants (*p* < 0.05) The frequency of eating biscuits and cakes was significantly associated with overweight or obesity among the students (χ2 ¼ 24.633, p ¼ 0.017). The type of milk often used (specifically full cream milk) was significantly associated with overweight and obesity among the students (χ2 ¼ 20.301, *p* < 0.05).
Adeomi et al. (35)	Dietary pattern The component (PC1) accounted for (35.3%) consumption of Fruits and vegetables, meat, poultry, egg and products, fish and milk. The component (PC2) accounted for (9%) consumption of carbohydrate and starchy foods, and tubers, while component (PC3) accounted for (7.5%) consumption of nuts and seeds, fats and oil, and condiments	Body Mass Index for age (WHO Anthro-plus) Overweight/obese −12.8%	Chi-square and cross tabulation The dietary component dominated by carbohydrate/starchy foods and roots and tubers foods were significantly associated with adolescent obesity (*p* = 0.033)
Mapfumo et al. (36)	Meal intake (breakfast) Majority consumed breakfast every morning (87.4%) and (53.2%) received at least three meals per day. Most frequently consumed meals were breakfast (87.4%) and supper (98.2%) Snack consumption Most (68.5%) of the children consumed snacks between meals	Body Mass Index (WHO Anthro-plus) Overweight/obesity – 5.4% Waist circumference Abdominal obesity – 13.5% Waist hip ratio Abdominal obesity 27.0% Waist to height ratio Abdominal obesity – 19.8%	Chi-square No significant association between breakfast, snack consumption and overweight/obesity status.
Olatona et al. (37)	Breakfast consumption More than half (56.7%) of the respondents consumed breakfast Majority (84.7%) ate breakfast at home and few (13.4%) ate breakfast at school The most common foods consumed as breakfast were bread and egg, followed by cereals and milk.	Body Mass Index for age (WHO Anthro-plus) Overweight −7.1% Obesity – 3.0%	Chi-square No association between breakfast intake and BMI status (*χ*^2^ = 2.412, *p* = 0.491) The mean BMI for those who skipped breakfast was significantly higher than that of those who ate breakfast (19. 33 ± 3. 27 kg/m2; 18.56 ± 3.05 kg/m2; *p* = 0.019)
Samuel et al., (38)	Snack consumption (In between meal) Majority of Overweight (65.9%) and obese (46.2%) subjects took in-between-meal snacks daily. Alcohol Consumption Majority (90%) of the subjects reported no or rare intake of alcohol.	Body Mass Index Overweight – 4.1% Obesity – 1.3%	Multiple logistic regression No association between intake of in between meal snacks and overweight/obesity No statistical significance was found between the differences in percentages of obesity and overweight in relation to the number of daily meals, fast food, vegetable and fruit consumption, intake of soft drinks and alcohol consumption.
Biadgilign et al. (39)	Soft drink consumption A good number of the respondents took soft drinks once and twice per week (29.1 and 30.2%), compare to those who took soft drink three time (6.6%) and four times and more per week (14.3%)	Body Mass Index for age (WHO Anthro-plus) Overweight – 57.7% Obesity – 53.9.9%	Multivariate logistic regression Children and adolescent who drank soft drinks for four or more times per were more likely to be overweight/obese compared with those who did not drink (AOR = 3.24; 95% CI 1.13, 7.95)
Elias et al. (40)	Fast-food consumption Some (32.2%) of the respondents ate fast food more than twice in the past week. Meanwhile, 23.8% did not eat any fast food, and 76.2% had it at least once in the past week.	Body Mass Index (WHO Anthro-plus) Overweight – 5.1% Obesity – 0.9%	Pearson correlation There is a significant connection between how often fast food is eaten and the likelihood of being overweight or obese. Multivariate logistic regression Adolescents who frequently ate fast food were almost three times more likely to be overweight or obese compared to those who ate fast food twice or less per week (AOR = 2.98; 95%CI: [1.89–4.76]).

### Nutritional status

- Overweight and obesity status of respondents

The prevalence of overweight and obesity among adolescents in Sub-Saharan Africa, as reported in the reviewed studies, varied considerably. Overweight prevalence ranged from 4.1 to 26%, and obesity prevalence ranged from 0.2 to 13.5% (22–40). A few studies also reported central obesity, using the waist-to-height ratio (WHtR) or waist-to-hip ratio (WHR) indicators, with rates of up to 27% in school-based populations (33). A noticeable pattern of gender differences in overnutrition was evident among the studies. Compared to males, adolescents aged 12 to 18 years consistently had higher rates of overweight and obesity among females (22, 27, 28, 31, 36). This trend may be explained by various overlapping factors, including differences in physical activity levels, sociocultural norms regarding body image, dietary behaviours such as meal skipping, and hormonal changes at puberty. For example, girls skip breakfast and exercise less, which contributes to excess weight gain (41).

Living in an urban area was also strongly associated with overnutrition. Adolescents in urban areas faced additional challenges, including greater access to fast-food outlets, processed snacks, and sugar-sweetened beverages, as well as fewer opportunities for physical activity due to limited open space and increased screen time. Another review found that adolescents in urban areas were more likely to have higher BMI and central adiposity than those in rural areas (25, 26, 29, 30, 32). These results underscore the critical importance of addressing the obesity epidemic among adolescents and, in particular, the rapid spread of obesity in urban and peri-urban obesogenic environments through effective urban policies and community approaches.

- Double burden of malnutrition

Although this review focuses primarily on the emerging challenge of overweight and obesity during adolescence, it is also important to recognise the growing burden of undernutrition that coexists in many low—and middle-income contexts. The double burden of malnutrition is the coexistence of undernutrition (stunting, wasting, and underweight) and overnutrition (overweight and obesity) within a population, often in the same communities or households.

In specific settings, such as rural and low-income areas, undernutrition still poses a significant threat. High rates of stunting among adolescents have been reported in several studies, ranging from 10.3 to 25.5% (32–36). For example, in a study by Otuneye et al. (36), who reported that 62.3% of orphaned children in Nigeria were stunted. These high rates indicate food insecurity, inadequate dietary diversity and limited access to nutrient-rich meals. On the other hand, while urban adolescents had access to significantly improved food availability, they often represented the other end of the malnutrition continuum, as their high consumption of energy-dense and nutrient-poor foods had led to overweight and obesity rates that approached 35% in contexts without fair food availability (22, 29, 36).

The dual burden was also reflected in gender disparities. Several studies showed that girls were more prevalent among overweight and obese children, while boys were more likely to be stunted or underweight (28–35). This gender gap in nutrition may be due to biological differences and social factors, including food allocation, cultural norms, and gendered practices related to physical activity.

This duality of nutrition in these studies highlights the multifaceted nature of adolescent health in Sub-Saharan Africa. It also directs interventions across multiple dimensions to provide food security and improve dietary quality and lifestyle behaviours, while considering the socioeconomic and cultural contexts that shape these adolescent experiences.

### Dietary patterns

- Meal skipping

Skipping meals, particularly breakfast, was also among the most prevalent behaviours reported in the studies reviewed. This was particularly common among urban adolescents; in some populations, more than one-fifth regularly skipped breakfast (27–29). This behaviour was significantly associated with higher odds of overweight and obesity. Thus, it could lead to impaired metabolic regulation, delayed signals of satiety, and a higher likelihood of compensatory snack consumption later in the day.

Multiple studies have shown a statistically significant inverse association between breakfast consumption and BMI. For example, Debeila et al. (33) reported that breakfast consumption among young people was associated with a 40% reduced possibility of being overweight or obese (AOR = 0.6; 95% CI: 0.34–0.97). Sedibe et al. (27) reported a significantly increased risk of being overweight (OR = 1.53; 95% CI = 1.10–2.13) among adolescents who reported skipping breakfast on weekdays. Gebremichael et al. (24) showed that adolescents who were reported to snack two to three times a day, often after skipping meals, were 26 times more likely to be overweight.

Alternatively, a few studies did not find a significant association between skipping breakfast and BMI, but did find a higher average BMI among breakfast skippers (31, 37). These inconsistencies may stem from variability in the definition of ‘breakfast’, differences in the types and quality of breakfast consumed, or differences in dietary intake methods (e.g., food frequency questionnaires versus 24-h recalls).

Notably, socioeconomic status emerged as a crucial factor influencing breakfast consumption (30, 35, 37). Low-income children and adolescents were more likely to miss breakfast due to limited access to food or insufficient funds. Thus, the practice of “absence” (in this case, of food) should be considered both a behavioural choice and a manifestation of broader structural inequities in food access.

- Discretionary food and beverage consumption

Frequent consumption of discretionary foods, those high in added sugars, saturated fats and refined carbohydrates, was singled out as a key factor in overweight and obesity. Several studies reported regular consumption of sugary beverages, fried foods, fast food, processed meats and sweetened snacks among children and adolescents (22, 26, 27, 29, 30, 33, 35). These dietary patterns indicate a transition from traditional, nutrient-rich foods to Western-style diets associated with globalisation and urbanisation.

Similarly, Alangea et al. (28) reported that children and adolescents with high-energy-dense dietary patterns (fried foods, processed meats, soft drinks) were more likely to be overweight. In another study, adolescents who consumed soft drinks ≥4 times/week had more than three times the odds of being overweight/obese (AOR = 3.24; 95% CI  = 1.13–7.95) (39). Also, Tunkara-Bah et al. (34) observed positive relationships between the intake of sweetened beverages and biscuits and rising BMI-for-age z-scores.

Although some adolescents had access to a wide variety of foods, that diversity was often of low quality. For instance, Adeomi et al. (32) showed that higher dietary diversity scores did not correlate with superior nutritional status when diets remained dominated by energy-dense, low-nutritional-quality foods. Such a distinction emphasises the importance of considering the quality and nutrient density of the foods we consume, as well as their diversity.

- School food environment

Food environments influence dietary behaviours in the school setting, where a large proportion of students’ daily food intake occurs. Nutrition policies or structured meal programs were often absent in many schools, both rural and urban, enabling the proliferation of vendors selling energy-dense, low-nutrient foods such as chips, snack cakes, and soda, as well as fried starchy foods (26, 27, 30–32). As a result, these foods became the go-to lunch for students and promoted unhealthy eating habits.

On the other hand, dietary and anthropometric progression in pupils was more significant in schools that implemented interventions related to food or meal programs. For example, Olatona et al. (37) reported a positive association between school meal programs emphasising fresh and minimally processed, local foods, children’s growth indices and a lower prevalence of overweight and obesity. Findings by Adeomi et al. (32) showed that schools without clear guidelines and supportive settings tended to have higher fat and calorie content, leading to poor food choices.

Again, socioeconomic inequalities were evident in the school context. Low-income adolescents also tended to attend schools without structured nutrition programs and had limited purchasing power, possibly leading them to buy mostly cheap junk food (26, 33). This emphasises the importance of policy changes in school food environments: nutrition education, vendor management, and low-cost, nutritious school meal options.

### Association between dietary patterns, overweight and obesity

This comprehensive review of studies shows that certain dietary behaviours are consistently associated with adolescents being overweight or obese. Although the magnitude and specificity of such associations differ across context, sex, and socioeconomic group, the directionality is evident: high-energy-dense, irregular, and low-nutritional-quality foods are consistently associated with excessive weight gain during adolescence.

- Fast food and ultra-processed foods

High intake of fast foods and ultra-processed foods was a consistent risk factor for overweight and obesity. Higher consumption of fast food (≥2 servings/week) was significantly associated with being overweight/obese in adolescents [adjusted odds ratio (AOR) = 2.98; 95% CI: 1.89–4.76] (30). One study found that an energy-dense dietary pattern (high intake of soft drinks, processed meat, fried foods, and pastries) was positively associated with overweight (AOR = 1.34; 95% CI = 1.04–1.70). These were supported by Tunkara-Bah et al. (34), who reported significant associations (*p* < 0.05) between sweetened snack intake, for example, biscuits, and increased BMI z-scores. These data indicate that frequent exposure to and consumption of highly palatable, energy-dense foods, particularly in urban and peri-urban contexts, are major drivers of calorie overconsumption and associated weight gain among adolescents.

- Soft drinks and sugary beverages

Sugar-sweetened beverages (SSB), such as soft drinks and sweetened fruit juice, were among the most consistently reported dietary contributors to overweight and obesity. Biadgilign et al. (39) found that adolescents who drank soft drinks four or more times a week had more than three times greater odds of being overweight or obese (AOR = 3.24; 95% CI = 1.13–7.95). Similarly, Tunkara-Bah et al. (34) found a significant association between the intake of soft drinks and higher BMI-for-age z-scores (*p* < 0.01). These drinks have been shown to replace healthier options such as water and milk while providing substantial “empty” calories that add to total energy intake in the day without significant satiety (34, 39).

- Meal skipping and irregular eating patterns

Irregular meal patterns, such as skipping breakfast, were associated with a greater risk of excess weight. In contrast, another study found that, on average, adolescents who skipped breakfast daily and ate more than 3 snacks per day were 10–26 times more likely to be overweight than those who maintained a regular eating pattern (24). Debeila et al. (33) found that regular breakfast consumption lowered the risk of obesity by 40% (AOR = 0.6; 95% CI = 0.34–0.97), whereas Sedibe et al. (27) performed an analysis of weekday breakfast skipping as a risk factor for overweight and reported increased odds of overweight among weekday breakfast skippers (OR = 1.53; 95% CI = 1.10–2.13).

Besides skipping meals, the timing of food intake seemed relevant. Late-night snacking was a major risk factor: adolescents who consumed snacks before bed had about 10 times the odds of being overweight than those who snacked earlier in the day (30). This approach is associated with a disturbed energy balance, dysfunctional glucose metabolism, and elevated total caloric intake.

- Dietary diversity and quality

Although dietary diversity is typically associated with better nutrition, some studies show a more complex relationship. The study by Adeomi et al. (32) showed that adolescents living in institutional care with moderate-to-high dietary diversity still exhibited high rates of undernutrition, suggesting that diversity alone does not guarantee intake of necessary nutrients. Similarly, Debeila et al. (33) observed that adolescents with high dietary diversity but frequent meal skipping or heavy consumption of discretionary foods also had increased BMI scores.

- Urban vs rural dietary influence

Urban and rural settings exhibit significant differences in adolescent dietary behaviours and outcomes. Urban adolescents tended to be more exposed to various fast foods, processed snacks, and sugary drinks, |and thus more susceptible to overweight and obesity (27, 29, 33). In contrast, though rural adolescents tended to have more traditional meals, they also suffered undernutrition with low dietary diversity and inadequate protein or calorie content (25, 26). Urban adolescents also had higher waist-to-height ratios and higher obesity prevalence, suggesting that environmental exposure is significant in shaping dietary exposure risk. Notably, greater urban nutrition knowledge among adolescents did not always lead to healthier eating behaviour, suggesting a gap between nutrition knowledge and practice (36).

- Protective dietary habits

Specific eating behaviours were consistently linked to healthier weight outcomes. Regular breakfast consumption, sufficient hydration (especially water), and daily fruit and vegetable intake were protective factors against overweight and obesity (25, 27, 33). For example, Debeila et al. (33) reported that adolescents who had a daily breakfast and regular fruit consumption had lower BMI. However, results were not entirely consistent across the studies. Few studies (31, 37) demonstrated that mean BMI values were lower among breakfast consumers, while the relationship between breakfast and obesity was statistically nonsignificant. However, these inconsistencies may be attributable to differences in cultural eating patterns, sample characteristics, or how data were collected (e.g., food frequency questionnaires vs. 24-h recalls) in these studies.

### Other key influencers of nutritional outcomes

While unhealthy dietary practices and habits are linked to increased weight gain, several factors can influence diet-related practices or directly impact nutritional status.

- Nutrition knowledge and awareness

Three studies involving school-going adolescents assessed nutrition-related knowledge (33, 36, 37). The studies employed pre-existing multiple-choice questionnaires, including well-validated, standardized, and institutionalized instruments (36, 37), as well as researcher-developed questions (33), either in their original or adapted forms. These instruments evaluated participants’ general nutrition knowledge and understanding of healthy eating principles (33, 36), while one study specifically assessed knowledge related to breakfast consumption (37).

Although the authors recognized nutrition knowledge as an important factor that may influence dietary behaviours and, consequently, weight-related outcomes, only one of the included studies (36) examined nutrition knowledge as a predictor of overweight and obesity.

- Socio-demographic influences on nutritional status

Socio-demographic factors have been shown to influence adolescents’ nutritional status in many studies. These factors include age, gender, socioeconomic status (SES), and parental occupation or education (22–37). It has been established that adolescents from rural areas or low SES areas have higher rates of undernutrition, like stunting and wasting, whereas those from urban areas are at an increased risk of overweight and obesity; this shows differences in nutritional status across various regions (25, 26, 28, 29, 31, 37). In a similar context, there are some gender differences where females are more likely to be overweight or obese, whereas males are more likely to be underweight (22, 27, 29, 31, 35, 36). Age is another important factor, as older adolescents tend to have poorer nutritional outcomes, as they have greater autonomy in selecting their meals and less dependence on parents (29, 30, 37). Moreover, parents’ education level and job status are important; adolescents whose parents hold professional jobs or are highly educated tend to eat better than those whose parents do menial jobs or have low educational qualifications (28, 31, 33, 36). These findings highlight the complex relationship between socio-demographic factors and adolescents’ nutritional well-being.

- Physical activity and sedentary behaviours

Nine included studies reported on physical activity and sedentary behaviour (22, 25, 29, 31, 33, 35, 36). Those studies have primarily used self-questionnaires and validated instruments to assess levels and timing of activity and sedentary time. For example, in two of the studies (27, 35) listed above, the International Physical Activity Questionnaire (IPAQ) was used. In contrast, one study employed the Physical Activity Questionnaire for Adolescents (PAQ-A) (31). Moreover, four studies reported sedentary behaviour, including screen time and prolonged sitting (22, 29, 31, 32). The findings showed that low physical activity levels are associated with a higher prevalence of overweight and obesity (27, 31, 33), and that urban adolescents are more sedentary than their rural counterparts (22, 29). On the other hand, walking and cycling to school, which are forms of active commuting, were protective against obesity in two studies (25, 32). Participation in sports and organised physical activities was found to be correlated with more favourable BMI and fitness levels in three studies (27, 31, 33). Thus, physical inactivity emerges as a prime risk factor for overweight and obesity, while increased physical activity remains positively associated with anthropometric outcomes (22, 25, 27, 29, 31, 33, 35, 36).

### Conceptual framework

**Figure 3:**
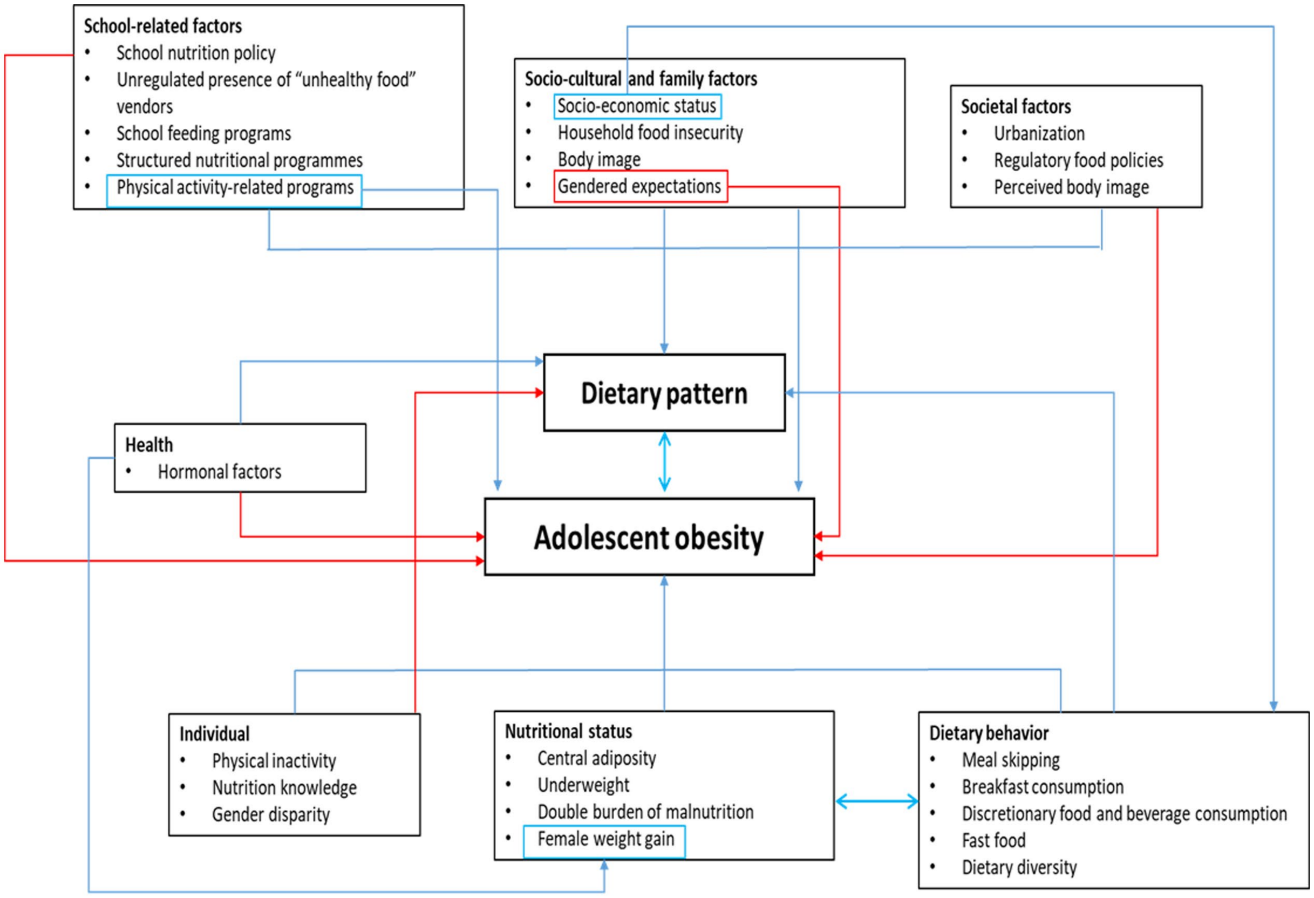
Conceptual framework illustrating the association between dietary factors and obesity. Adapted from Vilar-Compte et al. (42) under CC BY 4.0 and modified to reflect the objectives and variables of the present study.

The key themes depicted in this review are illustrated in the conceptual framework shown in Figure 3 (42). The blue lines indicate direct associations found in the reviewed studies, while the red lines indicate indirect associations observed. These connections have been summarized to help explain the multifaceted system surrounding childhood and adolescent obesity.

## Discussion

This systematic review reveals a consistent pattern of nutritional transition among children and adolescents in Sub-Saharan Africa, characterized by the rising obesity rate and a persistent burden of undernutrition. These findings align with the global literature documenting a shift toward energy-dense, nutrient-poor dietary habits, particularly among urban youth. Similar to previous studies (42, 43), our results confirm that urbanization and lifestyle changes significantly contribute to adolescent obesity through increased access to processed foods, decreased physical activity, and a growing dependence on fast food and sugary beverages. A plausible explanation is that children and adolescents spend much of their waking hours in school (12, 13), where food choices are often shaped by foods purchased with pocket money. In many African school settings, these options are dominated by readily available energy-dense, nutrient-poor foods and beverages (44, 45), potentially contributing to unhealthy dietary patterns and increasing overweight and obesity.

However, in contrast to studies from high-income countries, where structured school feeding programs and stronger regulatory food policies help reduce these risks, many of the sub-Saharan African contexts included in this review lacked such an institutional shield (46). The widespread absence of school nutrition policies and the unregulated presence of unhealthy food vendors common in sub-Saharan school environments suggest a policy gap that leaves adolescents vulnerable to poor dietary choices. This divergence highlights the need for context-specific interventions that account for infrastructure limitations and the region’s unique socioeconomic realities.

Gender disparities also stood out across studies, with girls consistently more likely to be overweight or obese, while boys were often more stunted or underweight. While this pattern is similar to findings from other low- and middle-income countries (LMICs), our review suggests that sociocultural and family-related norms regarding body image, household food distribution, and gendered expectations for physical activity may be amplifying these differences (41). Moreover, hormonal changes during puberty, combined with behaviours like breakfast skipping, appear to disproportionately impact adolescent girls.

This review also emphasizes the concern towards the double burden of malnutrition, the coexistence of under- and over-nutrition within the same community or household. This review showed that the double burden of malnutrition was particularly pronounced in rural areas, where food insecurity persists, and in urban settings, where dietary excess is more prevalent. While these findings mirror global patterns described by the WHO, our review adds regional specificity by demonstrating how socioeconomic status influences dietary behaviours such as meal skipping, not merely as a lifestyle choice, but often as a necessity rooted in food insecurity (47).

Meal skipping, particularly breakfast, was repeatedly linked to higher odds of obesity, emphasizing the findings of prior studies in both developed and developing countries (26, 27, 48–51). Yet, some inconsistencies were noted. A few studies did not find statistically significant associations between breakfast skipping and BMI, though higher mean BMI values were still observed among breakfast skippers. These inconsistencies likely stem from methodological differences, including how breakfast was defined or assessed, as well as the inherent limitations of dietary recall methods.

Consuming discretionary foods was another prominent theme. Our findings agree with those of earlier studies, which have shown that diets rich in sugary drinks, processed snacks, and fried foods are significantly associated with adolescent overweight (16, 52). Nevertheless, this review revealed an interesting contrast regarding dietary diversity. While often considered a marker of nutritional adequacy, dietary diversity in this context translates to a variety of low-nutrient, high-calorie foods. This suggests that dietary quality, not just diversity, must be prioritized in both public messaging and intervention design.

Overall, the evidence presented in this review highlights that child and adolescent malnutrition in Sub-Saharan Africa is driven by interconnected social, economic, and cultural determinants, including rapid urbanization, socioeconomic inequalities, changing dietary norms, and increasingly obesogenic food environments. These factors shape food choices, dietary behaviours, and access to nutritious foods, contributing to both undernutrition and overweight/obesity. The findings underscore the need for multisectoral interventions that extend beyond individual behaviour change to address structural determinants of nutrition. Policy actions should prioritize improving school food environments, regulating the availability and marketing of unhealthy foods and beverages, strengthening nutrition education, and enhancing access to affordable, nutritious foods. Such measures could inform context-specific strategies to improve adolescent nutrition outcomes and reduce the long-term burden of diet-related diseases across the region.

## Conclusion

The findings from this review emphasize the need for all-inclusive interventions targeting paediatric nutrition. Policies and programs should prioritize promoting healthy dietary patterns, focusing on consuming energy-dense foods and encouraging the intake of fruits, vegetables, and fibre. In addition, tailored interventions that account for geographic, socio-economic, and gender-specific differences, and the planning and implementation of school- and community-based initiatives to reduce sedentary behaviours and promote active lifestyles, would significantly mitigate the problem of Obesity amongst these adolescents. By addressing these factors holistically, stakeholders can help mitigate the double burden of malnutrition and promote healthier outcomes for adolescents across Africa.

## Strengths and limitations

This review has several strengths. It addresses an important and underexplored public health issue in Sub-Sharan Africa, incorporates studies from multiple African countries, follows PRISMA reporting guidance, and includes a registered PROSPERO protocol.

However, several limitations should be acknowledged. The included studies were predominantly cross-sectional, limiting casual inference. Variability in dietary assessment methods and anthropometric standards reduced comparability across findings. Additionally, no quantitative meta-analysis was conducted due to heterogeneity in study designs and outcome measures.

## Data Availability

The original contributions presented in the study are included in the article/Supplementary material, further inquiries can be directed to the corresponding author.

## Glossary

PRISMA: Preferred Reporting Items for Systematic Reviews and Meta-Analysis
AJOL: African Journals Online
BMI: Body Mass Index
PEO: Population, Exposure, Outcome
JBI: Joanna Briggs Institute
IBM SPSS: International Business Machines, Statistical Package for the Social Sciences
WHO: World Health Organization
WHR: Waist Hip Ratio
WHtR: Waist Height ratio
IOTF: International Obesity Task Force
FAO: Food and Agriculture Organization
MUAC: Mid Upper Arm Circumference
AHDSS: Agincourt Health and Demographic Surveillance System
FFQ: Food Frequency Questionnaire
BMA: Body Mass Index for Age
DD: Dietary Diversity
DDS: Dietary Diversity Score
CDC: Central for Disease Control and Prevention
IASO: International Association for the Study of Obesity
ANOVA: Analysis of Variance
SES: Socio-economic Status
IPAQ: International Physical Activities for Adolescents
PAQ-A: Physical Activities Questions for Adolescents
