# Supplementary material for: Linking diet and obesity among young people living in Sub-Saharan Africa: a systematic review
Source: Front Nutr. 2026 Jul 21;13:1859486. doi: 10.3389/fnut.2026.1859486 (PMC13433201; doi:10.3389/fnut.2026.1859486)
Supplement: Supplementary file 1 [file Table_1.DOCX]

**Supplementary File**

**Table 1. PubMed Search Strategy for diet and obesity**

| Search Terms | Hits |
| --- | --- |
| Anthropometric outcome |  |
| ("Obesity"[Mesh]) OR (Obese OR overweight OR weight gain OR body mass index OR BMI) | 927,778 |
| Dietary exposure |  |
| (Diet"[Mesh]) OR (dietary patterns OR diet OR dietary intake OR food consumption OR eating habits OR breakfast consumption OR fast-food consumption) | 1,120,758 |
| Children |  |
| ((Child"[Mesh]) OR (Adolescent"[Mesh])) OR (child OR adolescent OR youth OR school aged children OR young Children) | 4,892,709 |
| Africa |  |
| (sub-saharan africa[Mesh]) OR (africa OR nigeria OR zimbabwe OR Gambia OR ethiopia OR south africa) | 658,551 |
| S((((("Obesity"[Mesh]) OR (Obese OR overweight OR weight gain OR body mass index OR BMI))) AND (Diet OR (dietary patterns OR diet OR dietary intake OR food consumption[Title/Abstract] OR eating habits OR breakfast consumption[Title/Abstract] OR fast food consumption AND OR Adolescent OR child OR adolescent OR youth OR school aged children OR young Children AND (sub-saharan africa OR africa OR nigeria OR zimbabwe OR Gambia OR ethiopia OR south africa) | 605 |

**Search strings for Anthropometric outcome AND Dietary exposure AND children**

("Obesity"[MeSH Terms] OR ("obeses"[All Fields] OR "Obesity"[MeSH Terms] OR "Obesity"[All Fields] OR "obese"[All Fields] OR "obesities"[All Fields] OR "obesity s"[All Fields] OR ("overweight"[MeSH Terms] OR "overweight"[All Fields] OR "overweighted"[All Fields] OR "overweightness"[All Fields] OR "overweights"[All Fields]) OR ("weight gain"[MeSH Terms] OR ("weight"[All Fields] AND "gain"[All Fields]) OR "weight gain"[All Fields]) OR ("body mass index"[MeSH Terms] OR ("body"[All Fields] AND "mass"[All Fields] AND "index"[All Fields]) OR "body mass index"[All Fields]) OR "BMI"[All Fields])) AND ("Diet"[Title/Abstract] OR ("dietary patterns"[Title/Abstract] OR "Diet"[Title/Abstract] OR "dietary intake"[Title/Abstract] OR "food consumption"[Title/Abstract] OR "eating habits"[Title/Abstract] OR "breakfast consumption"[Title/Abstract] OR "fast food consumption"[Title/Abstract])) AND ("Child"[Title/Abstract] OR "Adolescent"[Title/Abstract] OR "Child"[Title/Abstract] OR "Adolescent"[Title/Abstract] OR "youth"[Title/Abstract] OR "school aged children"[Title/Abstract] OR "young children"[Title/Abstract]) AND ("africa south of the sahara"[MeSH Terms] OR ("africa"[All Fields] AND "south"[All Fields] AND "sahara"[All Fields]) OR "africa south of the sahara"[All Fields] OR ("sub"[All Fields] AND "saharan"[All Fields] AND "africa"[All Fields]) OR "sub-Saharan africa"[All Fields] OR ("africa"[MeSH Terms] OR "africa"[All Fields] OR "africa s"[All Fields] OR "africas"[All Fields]) OR ("nigeria"[MeSH Terms] OR "nigeria"[All Fields] OR "nigeria s"[All Fields]) OR ("zimbabwe"[MeSH Terms] OR "zimbabwe"[All Fields] OR "zimbabwe s"[All Fields]) OR ("gambia"[MeSH Terms] OR "gambia"[All Fields] OR "gambia s"[All Fields]) OR ("ethiopia"[MeSH Terms] OR "ethiopia"[All Fields] OR "ethiopia s"[All Fields]) OR ("south africa"[MeSH Terms] OR ("south"[All Fields] AND "africa"[All Fields]) OR "south africa"[All Fields])) AND ((humans[Filter]) AND (2000:2024[pdat]) AND (english[Filter]) AND (adolescent[Filter] OR child[Filter] OR preschoolchild[Filter]))

**Table 2. Study-Level Risk of Bias Assessment Across Individual Critical Appraisal Criteria**

| **Included Reviews** | **Inclusion criteria clearly defined** | **Study subjects and settings described in detail** | **Exposure measured in a valid and reliable way** | **Objective, standard criteria used for measurement** | **Outcomes measured in a valid and reliable way** | **Appropriate statistical analysis** | **Number of Criteria met** |
| --- | --- | --- | --- | --- | --- | --- | --- |
| Kristen *et al*., (22) | Y | Y | Y | Y | Y | Y | 6 |
| Teshome *et al*., (23) | Y | Y | Y | Y | Y | Y | 6 |
| Gebremichael and Chere, (24) | Y | Y | N | Y | Y | Y | 5 |
| Lateef *et al*., (25) | N | Y | Y | Y | Y | Y | 5 |
| Otuneye *et al.*(26) | Y | Y | N | Y | Y | Y | 5 |
| Alangae *et al*., (28) | N | Y | Y | Y | Y | Y | 5 |
| Sedibe *et al*., (27) | N | Y | Y | Y | Y | Y | 5 |
| Tluway *et al*.,(29) | N | Y | Y | Y | Y | Y | 5 |
| Gyamfi *et al*., (30) | N | Y | N | Y | Y | Y | 4 |
| Annan *et al*., (31) | Y | Y | U | Y | Y | Y | 5 |
| Adeomi *et al*., (32) | N | Y | Y | Y | Y | Y | 5 |
| Tukura-Bah *et al*., (34) | Y | Y | Y | Y | Y | Y | 6 |
| Debeila *et al*.,(33) | Y | Y | Y | Y | Y | Y | 6 |
| Adeomi *et al*., (35) | Y | Y | Y | Y | Y | Y | 6 |
| Mapfumo *et al*., (36) | Y | Y | N | Y | Y | Y | 5 |
| Olatona *et al*.,(37) | N | Y | NA | Y | Y | Y | 4 |
| Samuel *et al*., (38) | Y | Y | NA | Y | Y | Y | 5 |
| Biadgilign *et al*., (39) | Y | Y | Y | Y | Y | Y | 6 |
| Elias *et al*., (40) | Y | Y | Y | Y | Y | Y | 6 |

**Legend**

Y = Yes (criterion met)

N = No (criterion not met)

U = Unclear (insufficient information reported)

NA = Not Applicable.

Studies were rated as “Unclear” when insufficient information was provided in the original article to determine whether the criterion was met.
